# Supplementary material for: Amoeba-like self-oscillating polymeric fluids with autonomous sol-gel transition
Source: Nat Commun. 2017 Jul 13;8:15862. doi: 10.1038/ncomms15862 (PMC5511347; doi:10.1038/ncomms15862)
Supplement: Supplementary Information [file ncomms15862-s1.pdf]

Type of file: PDF
Size of file: 0 KB
Title of file for HTML: Supplementary Information
Description: Supplementary Figures, Supplementary Tables and Supplementary Discussion

Type of file: MOV
Size of file: 0 KB
Title of file for HTML: Supplementary Movie 1
Description: Observation of an autonomous sol-gel oscillation of an ABC triblock copolymer solution (5.0 wt%) at 30 °C. The fluidity of the solution was clearly oscillated repeatedly under constant condition coupled with the redox change of the Ru(bpy)3 during the BZ reaction. The BZ substrates in the feed for the measurements were composed as follows: [HNO3] = 0.81 M, [NaBrO3] = 0.1 M, [MA] = 0.04 M. The movie speed is 3× actual speed.

Type of file: MOV
Size of file: 0 KB
Title of file for HTML: Supplementary Movie 2
Description: Observation of amoeba-like intermittent forward motion of a polymer solution droplet (5.0 wt%) inside a tilted glass capillary at 26 °C. In order to clearly observe the sol-gel oscillation as a change in motility, the polymer solution and the substrate were mixed and immediately enclosed in the capillary. The BZ substrates in the feed for the measurements were composed as follows: [HNO3] = 0.81 M, [NaBrO3] = 0.1 M, [MA] = 0.04 M. The movie is presented at five times the actual speed.

Type of file: pdf
Size of file: 0 KB
Title of file for HTML: Peer Review File
Description:

Supplementary Discussion

As discussed in the main text, the oscillation period (T) can be expressed using the following empirical relationship for the concentrated ABC triblock copolymer solution:

0.1 wt%: $T = 1.78[HNO_3]^{-0.785}[NaBrO_3]^{-0.702}[MA]^{-0.415}$ (1)

5.0 wt%: $T = 1.50[HNO_3]^{0.624}[NaBrO_3]^{-0.515}[MA]^{-0.955}$ (2)

Here, we will discuss the reason why the oscillation period dependency against [HNO_3] shows opposite tendency depending on the [Polymer]. We compare the waveforms of the oscillation in each concentration condition to elucidate the factors affecting opposite dependency. FKN mechanism could be divided into three process, i.e., process A (consumption of bromide ion), process B (oxidation of $Ru(bpy)_3^{2+}$, autocatalytic reaction), and process C (reduction of $Ru(bpy)_3^{3+}$, production of bromomalonic acid) as shown below;

FKN mechanism

Process A (consumption of bromide ion)

$Br^- + HOBr + H^+$	⇄	$Br_2 + H_2O$	(1)
$Br^- + HBrO_2 + H^+$	⇄	$2HOBr$	(2)
$Br^- + BrO_3^- + 2H^+$	⇄	$HOBr + HBrO_2$	(3)

Process B (oxidation of $Ru(bpy)_3^{2+}$, autocatalytic reaction)

$2HBrO_2$	⇄	$HOBr + BrO_3^- + H^+$	(4)
$HBrO_2 + BrO_3^- + H^+$	⇄	$2BrO_2\cdot + H_2O$	(5)
$BrO_2\cdot + Ru(bpy)_3^{2+} + H^+$	⇄	$HBrO_2 + Ru(bpy)_3^{3+}$	(6)

Process C (reduction of $Ru(bpy)_3^{3+}$, production of bromomalonic acid)

$BrO_2 + Ru(bpy)_3^{3+} + H_2O$	⇄	$BrO_3^- + Ru(bpy)_3^{2+} \rightleftarrows 2H^+$	(7)
$Br_2 + CH_2(COOH)_2$	→	$BrCH(COOH)_2 + Br^- + H^+$	(8)
$6Ru(bpy)_3^{3+} + CH_2(COOH)_2 + 2H_2O$	→	$6Ru(bpy)_3^{2+} + HCOOH + 2CO_2 + 6H^+$	(9)
$4Ru(bpy)_3^{2+} + BrCH(COOH)_2 + 2H_2O$	→	$4Ru(bpy)_3^{2+} + HCOOH + Br^- + 2CO_2 + 5H^+$	(10)

By separation of waveform of BZ reaction oscillation period in each process (**Supplementary Fig. 12**), we could describe each process as shown in **Supplementary Table 2**.

Here, for simplicity, we assume that reaction kinetics of process B involving autocatalytic reaction characterizing BZ reaction is not changed by changes in the BZ reaction condition. In 0.1 wt% polymer solution, process A was accelerated while the duration of process C was not changed so much by increasing [HNO_3] from 0.60 M to 0.81 M[3]. As a result, a total oscillation period became short. In contrast, in 5.0 wt% polymer solution, the duration of process A became shorter, whereas that of process C did longer with increases in [HNO_3] from 0.70 M to 0.90 M. Therefore, the difference in the H^+ dependency on the process C is essential. Note that the total amount of [$Ru(bpy)_3$] in the solution linearly depends on the [Polymer].

In 0.1 wt% polymer solution, [$Ru(bpy)_3$] was diluted and [H^+] was saturated against [$Ru(bpy)_3$]. In other words, reaction kinetics of process C is determined by the [$Ru(bpy)_3$] introduced. This is the reason why process C was almost constant even if the [H^+] increased. On the other hand, process A, in which $Ru(bpy)_3$ does not participate the reaction, was consistently accelerated with increases in [H^+] in terms of chemical equilibrium (H^+, BrO_3^-, and other reagents in solution). Consequently, the total period governed by process A being shorter with increases in [HNO_3].

In 5.0 wt% polymer solution, we have to close look at the duration of the process C occupied in a period because [$Ru(bpy)_3$] was no longer diluted against [H^+] and sufficient amount of [$Ru(bpy)_3$] seemed to be supplied. In this case, H^+ is involved in equation (7)-(10) in the right-hand side in process C, thus the kinetics of these reactions must be reduced. As a result, process C was significantly shortened with increases in [HNO_3]. In summary, the period was increased with increases in [HNO_3] because the deceleration of process C was dominant rather than the acceleration of process A.

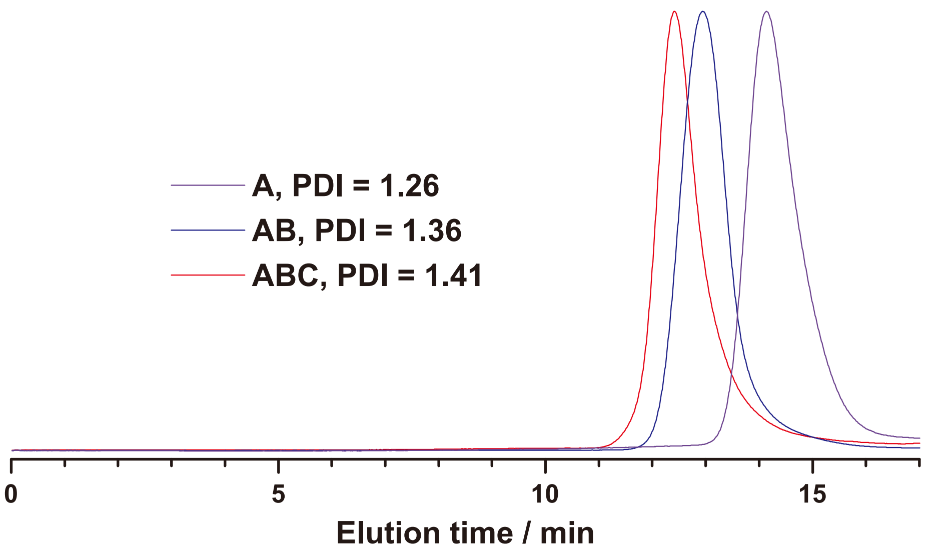

Supplementary Figure 1 GPC traces of the ABC triblock copolymer and its precursors synthesized in this study. (A: P(NIPAAm-*r*-BA), B: PDMAAm, C: P(NIPAAm-*r*-NAPMAm))

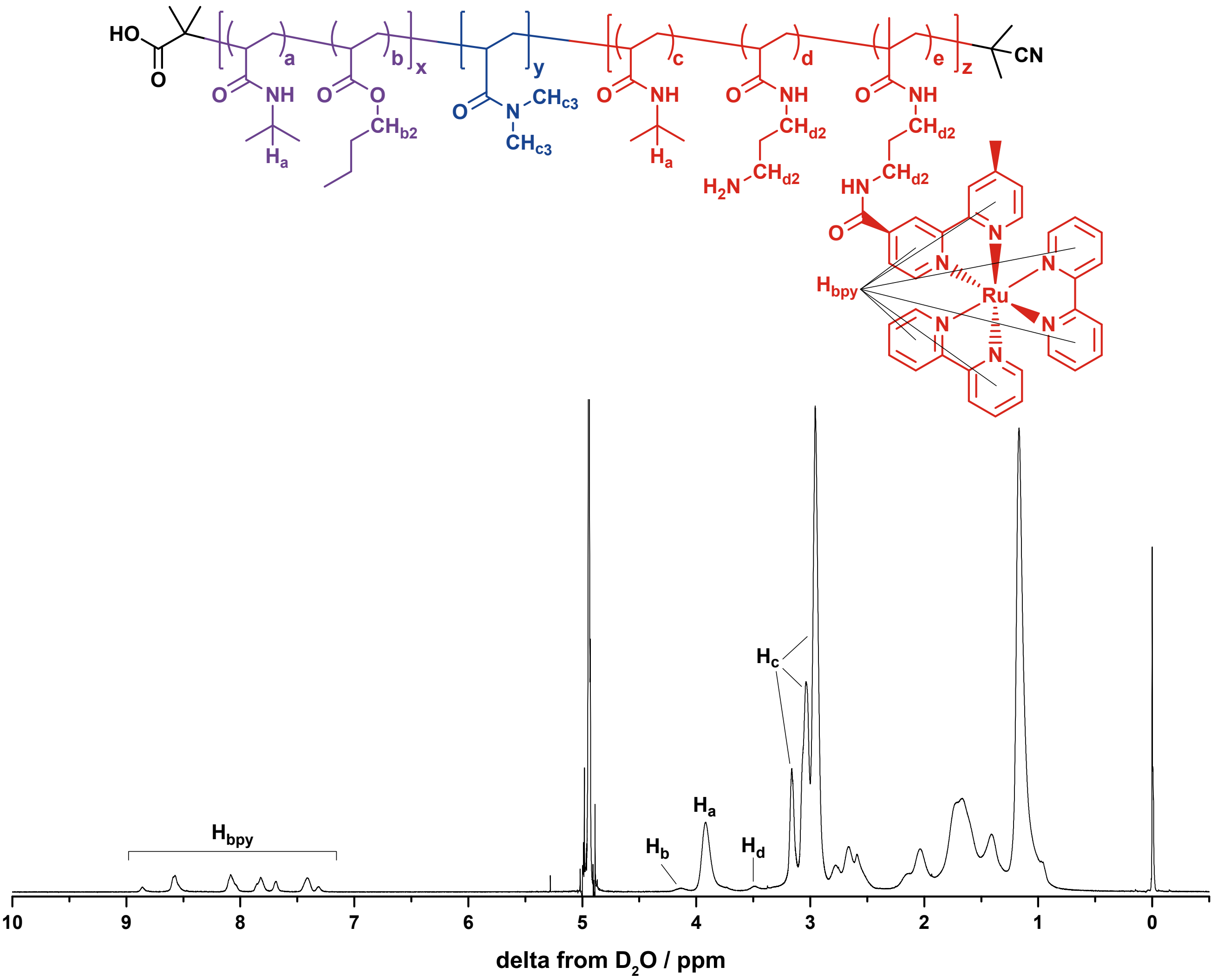

Supplementary Figure 2 ^{1}**H-NMR spectrum of the ABC triblock copolymer.**

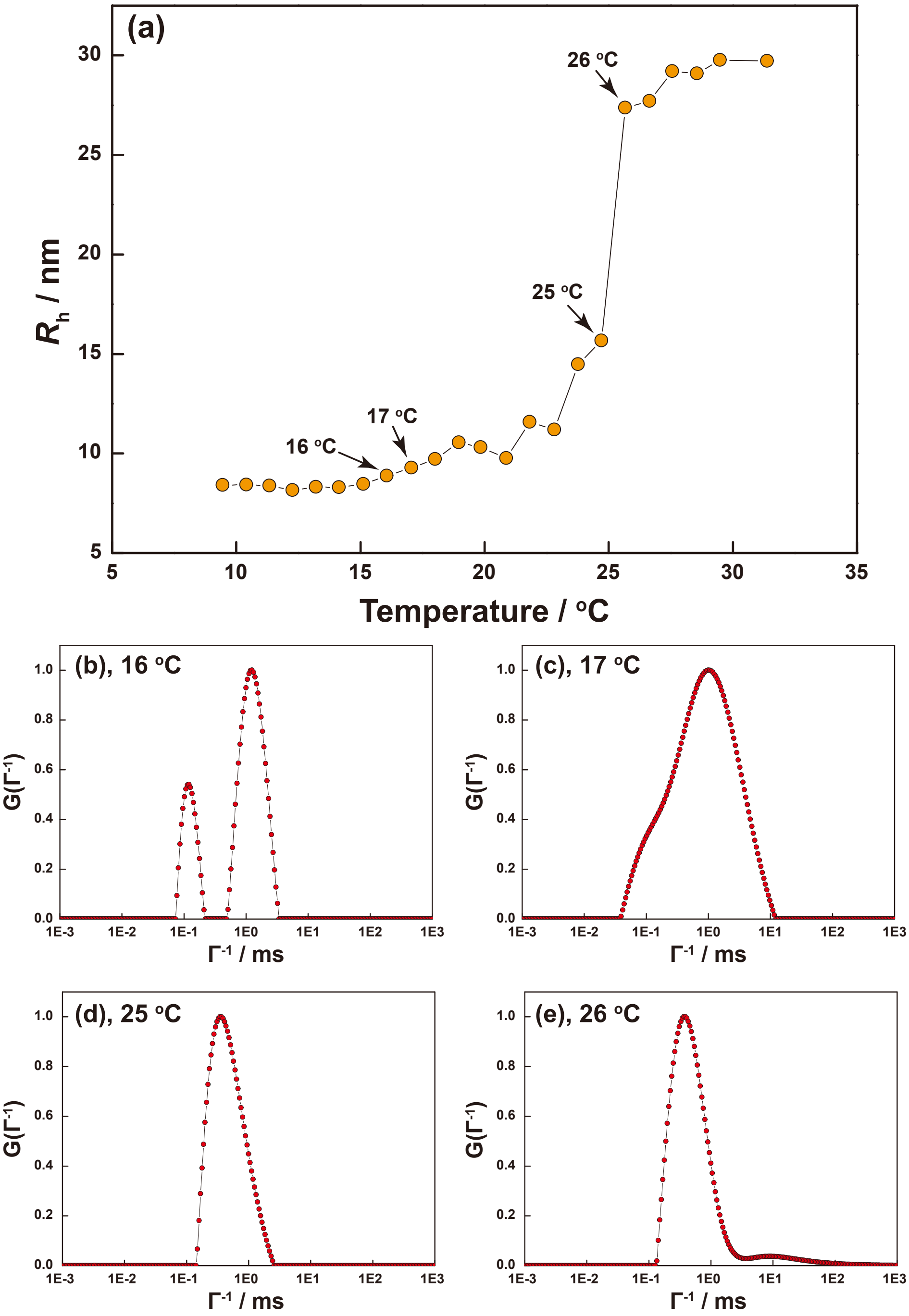

Supplementary Figure 3 Dynamic light scattering measurement of dilute polymer solution under equilibrium. **(a)** Temperature dependence of the hydrodynamic radius (R_h). **(b-e)** Decay time distribution function ($G(\Gamma^{-1})$) at **(b)** 16 °C, **(c)** 17 °C, **(d)** 25 °C, and **(e)** 26 °C of the ABC triblock copolymer (0.5 wt%) in the reduced state. The reduced state was maintained by adding 0.81 M HNO_3 and 0.1 M NaCl. The correlation function was collected via 30 s laser irradiation.

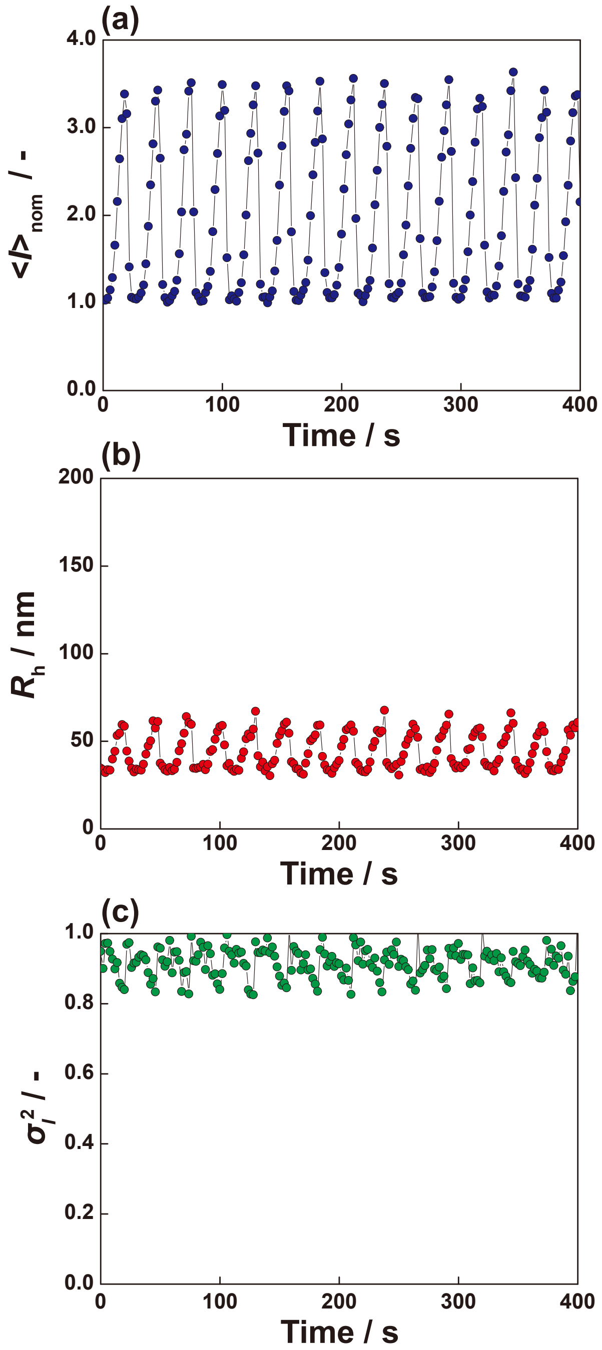

Supplementary Figure 4 Time-resolved dynamic light scattering measurement of 0.1 wt% dilute polymer solution under non-equilibrium. Oscillating profiles of **(a)** normalized scattering intensity, $<I>_{nom}$, **(b)** R_h, and **(c)** initial amplitude of the intensity correlation function, $\sigma_I{}^2$ during the BZ reaction for the ABC triblock copolymer solution (0.1 wt%) at 26 °C. The BZ substrates in the feed for the measurements were composed as follows: $[HNO_3]$ = 0.81 M, $[NaBrO_3]$ = 0.1 M, [MA] = 0.04 M. The correlation function was collected via 2.0 s laser irradiation.

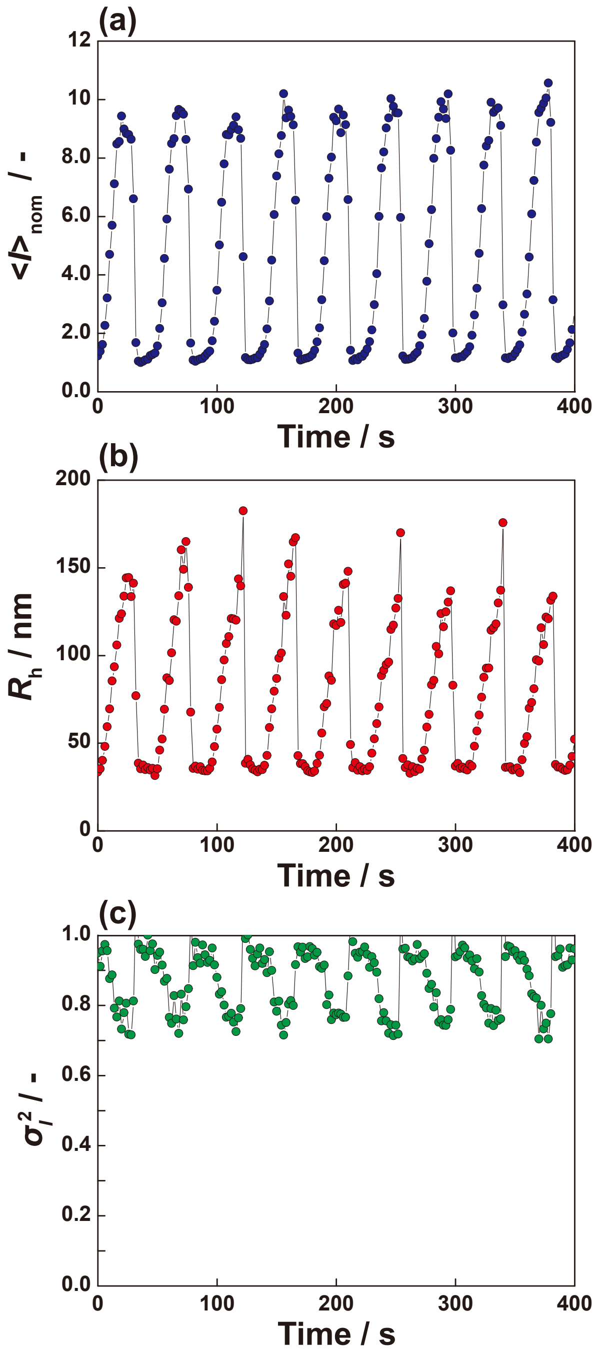

Supplementary Figure 5 Time-resolved dynamic light scattering measurement of 0.5 wt% dilute polymer solution under non-equilibrium. Oscillating profiles of **(a)** normalized scattering intensity, $\langle I \rangle_{nom}$, **(b)** R_h, and **(c)** initial amplitude of the intensity correlation function, $\sigma_I{}^2$ during the BZ reaction for the ABC triblock copolymer solution (0.5 wt%) at 26 °C. The BZ substrates in the feed for the measurements were composed as follows: [HNO_3] = 0.81 M, [$NaBrO_3$] = 0.1 M, [MA] = 0.04 M. The correlation function was collected via 2.0 s laser irradiation.

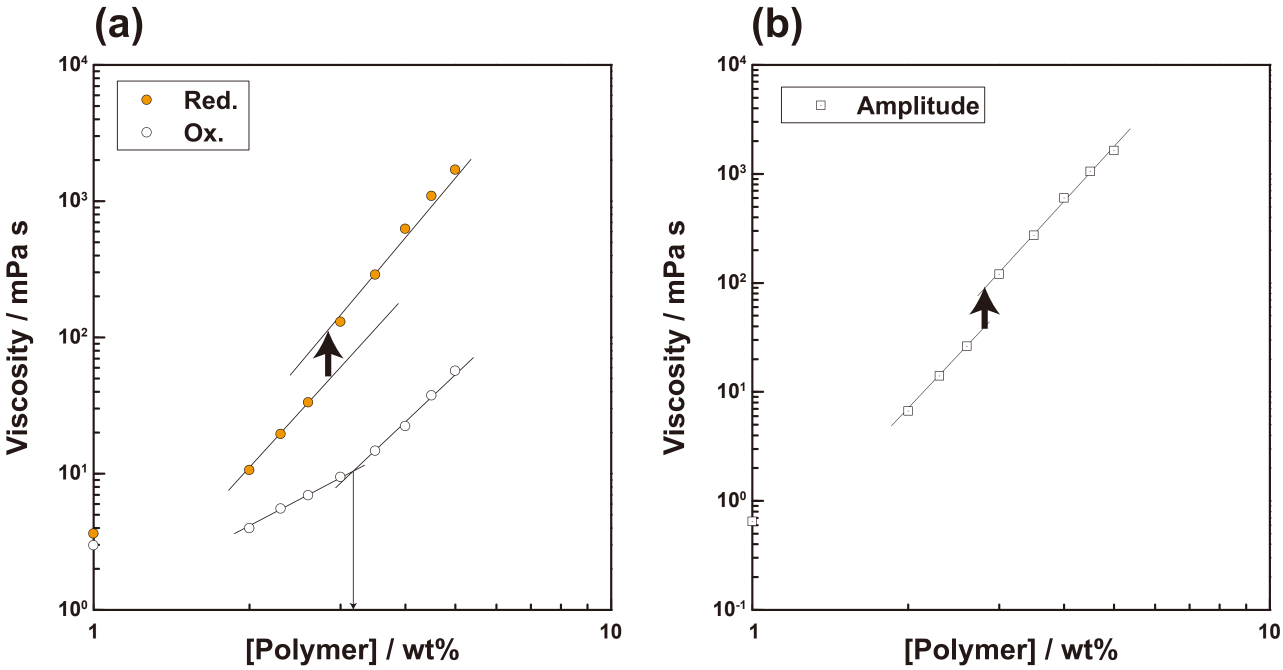

Supplementary Figure 6 (a) Polymer concentration dependence of the maximum (in the reduced state) and the minimum (in the oxidized state) viscosity values during the oscillation of the ABC triblock copolymer solutions, and (b) amplitude of the viscosity oscillation at 26 °C. The BZ substrates in the feed for the measurements were composed as follows: [HNO_3] = 0.81 M, [$NaBrO_3$] = 0.1 M, [MA] = 0.04 M, share rate = 45 s^{-1}.

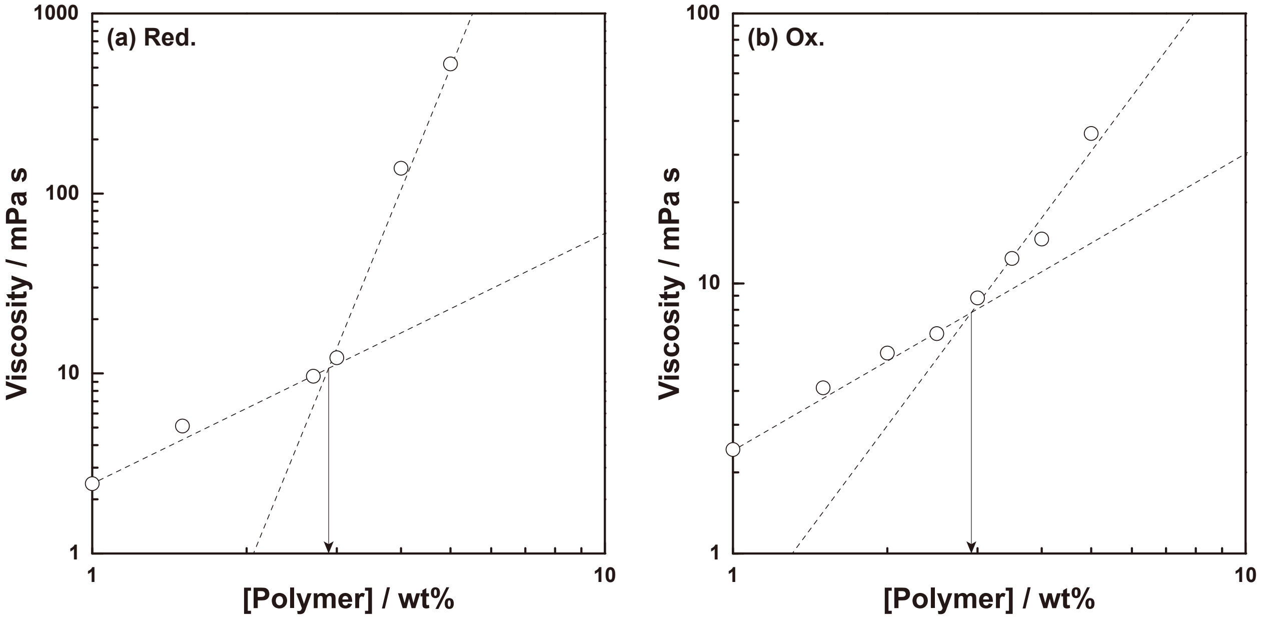

Supplementary Figure 7 Relationship between viscosity and polymer concentration in (a) the reduced ($Ru(bpy)_3^{2+}$), and (b) the oxidized ($Ru(bpy)_3^{3+}$) states for the ABC triblock copolymers. Each state was maintained by adding 0.81 M HNO_3 and 0.1 M NaCl and, 0.81M HNO_3 and 0.1 M $NaBrO_3$, respectively. Temperature = 26 °C, share rate = 45 s^{-1}.

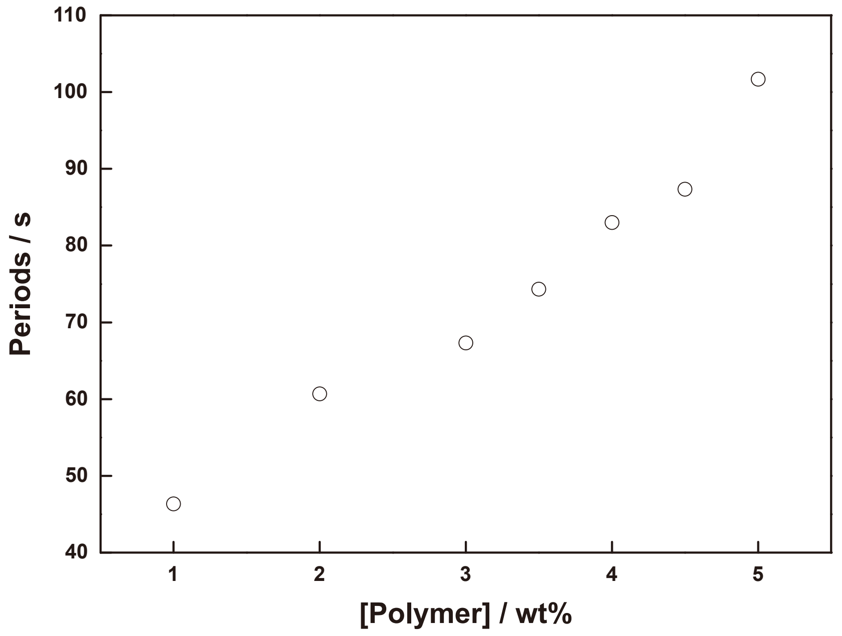

Supplementary Figure 8 Polymer concentration dependence of the oscillation periods for the ABC triblock copolymer solutions at 26 °C. The BZ substrates in the feed for the measurements were as composed follows: [HNO_3] = 0.81 M, [$NaBrO_3$] = 0.1 M, [MA] = 0.04 M, share rate = 45 s^{-1}.

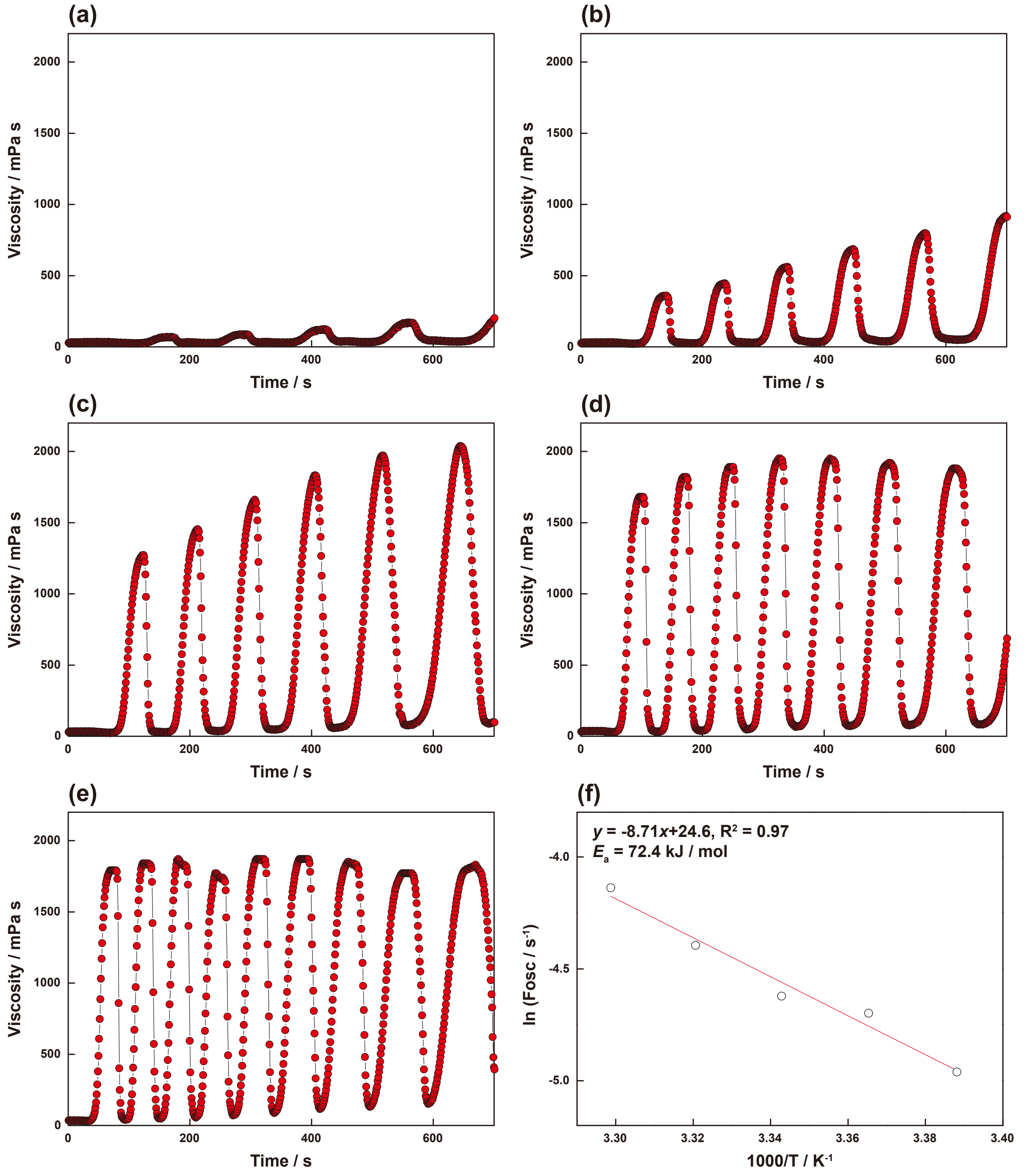

Supplementary Figure 9 Temperature dependence of the viscosity oscillation for the ABC triblock copolymer solutions (5.0 wt%). Temperatures of each measurements were set at **(a)** 22 °C, **(b)** 24 °C, **(c)** 26 °C, **(d)** 28 °C, and **(e)** 30 °C, respectively. **(f)** Arrhenius plot of the frequency of the oscillation (inverse of oscillation period), F_{osc}. The BZ substrates in the feed for the measurements were composed as follows: $[HNO_3]$ = 0.81 M, $[NaBrO_3]$ = 0.1 M, [MA] = 0.04 M, share rate = 45 s^{-1}.

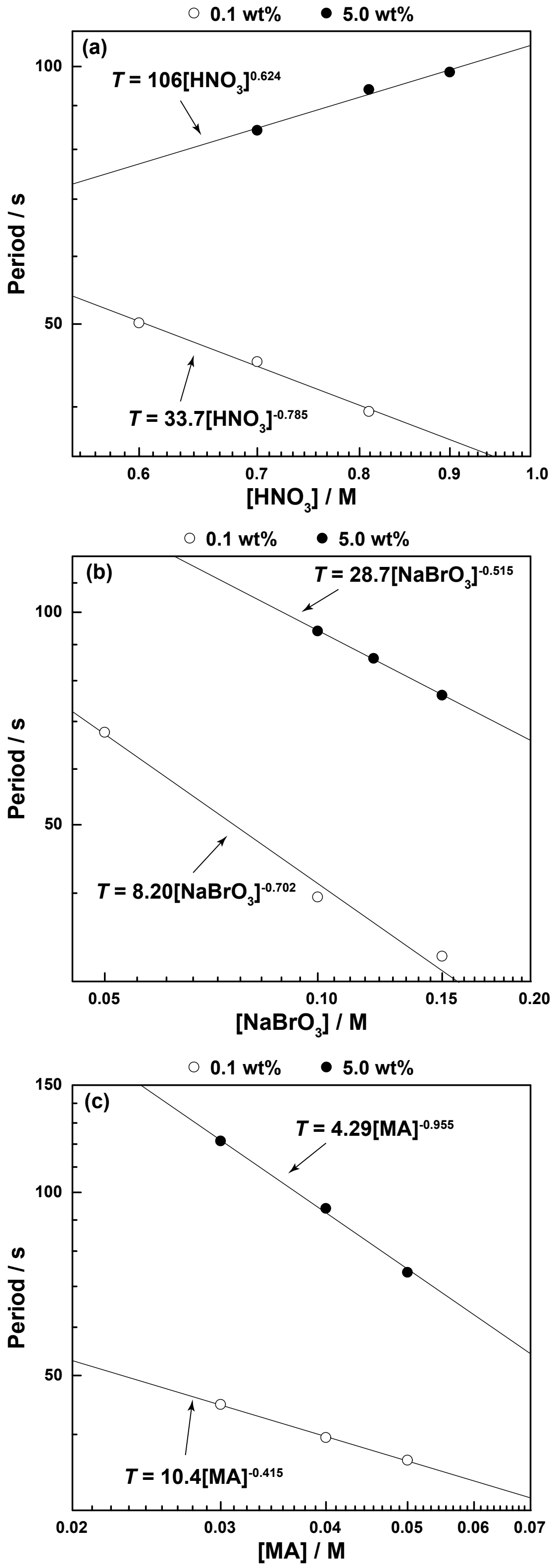

Supplementary Figure 10 Plots of oscillation period against initial concentration of a substrate under fixed initial concentration of the other substrate constituents for the 0.1 wt% and 5.0 wt% polymer solutions at 26 °C.

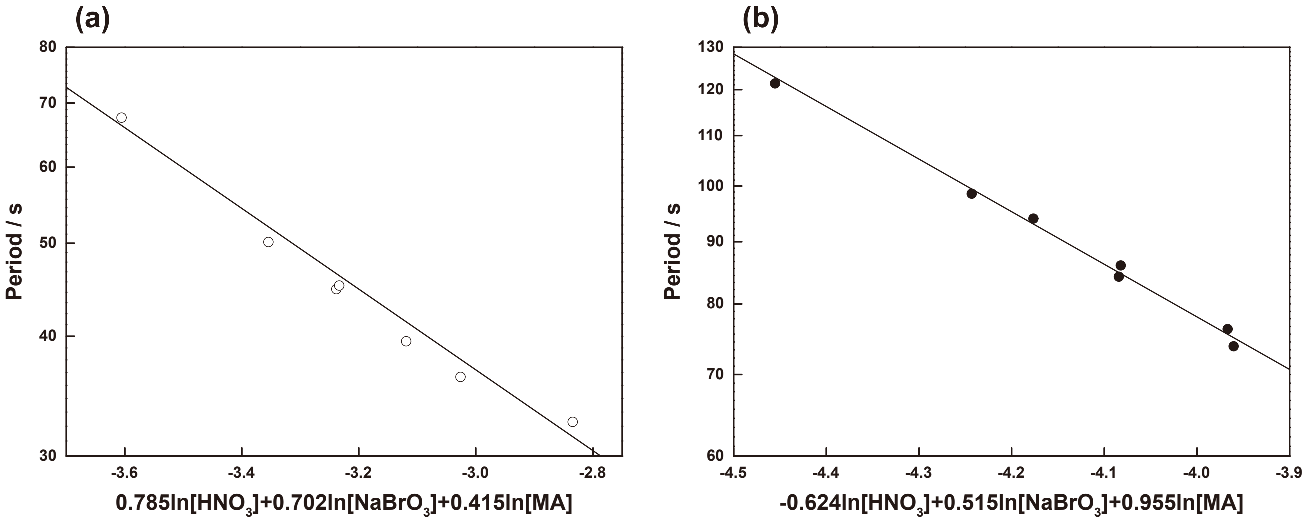

Supplementary Figure 11 Oscillating periods as a function of the initial concentrations of HNO_3, $NaBrO_3$, and MA for (a) the 0.1 wt% solution and (b) the 5.0 wt% solution.

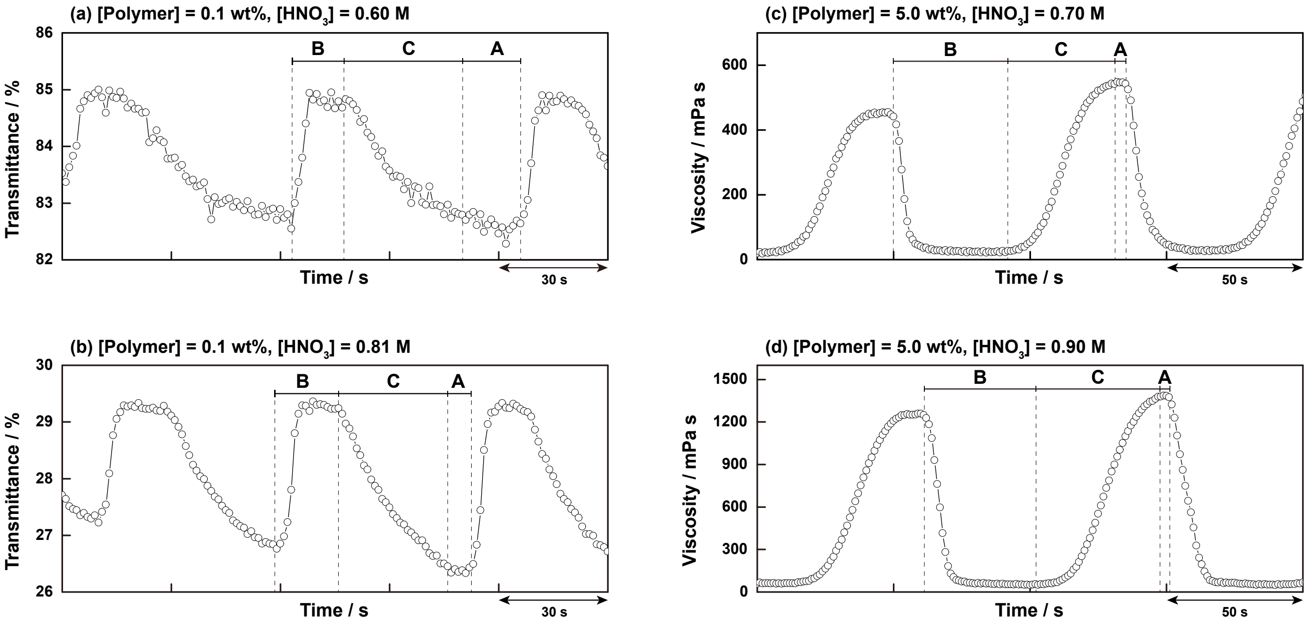

Supplementary Figure 12 Waveform analysis of ABC triblock copolymer solution at 26 °C. The BZ substrates in the feeds for the measurements were summarized in **Supplementary Table 2**.

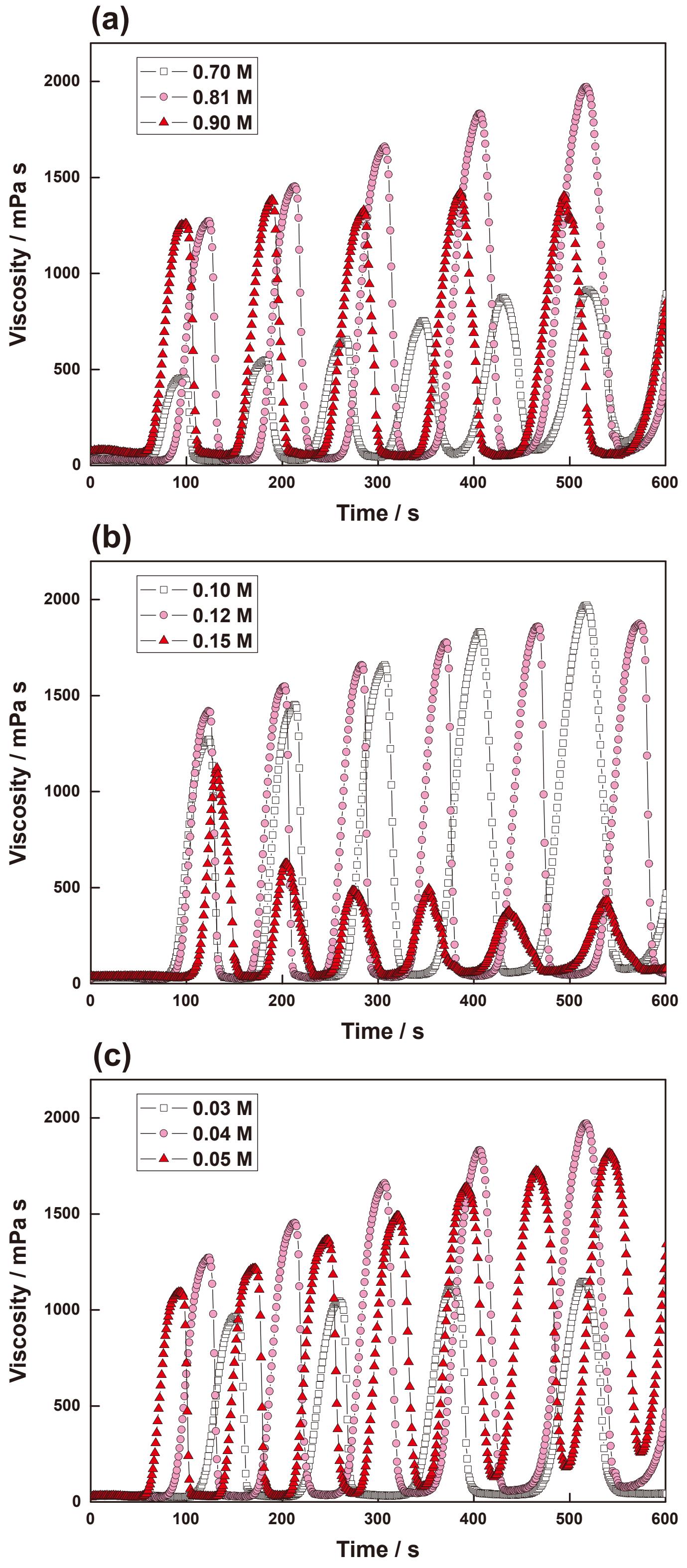

Supplementary Figure 13 Oscillating profiles of the viscosity for the ABC triblock copolymer solutions (5.0 wt%) at 26 °C under various BZ substrates conditions. The BZ substrates in the feed for the measurements were composed as follows: **(a)** $[HNO_3]$ = 0.70, 0.81, 0.90 M, $[NaBrO_3]$ = 0.1 M, [MA] = 0.04 M, **(b)** $[HNO_3]$ = 0.81 M, $[NaBrO_3]$ = 0.10 M, 0.12 M, 0.15 M, [MA] = 0.04 M, **(c)** $[HNO_3]$ = 0.81 M, $[NaBrO_3]$ = 0.1 M, [MA] = 0.03 M, 0.04 M, 0.05 M, share rate = 45 s^{-1}.

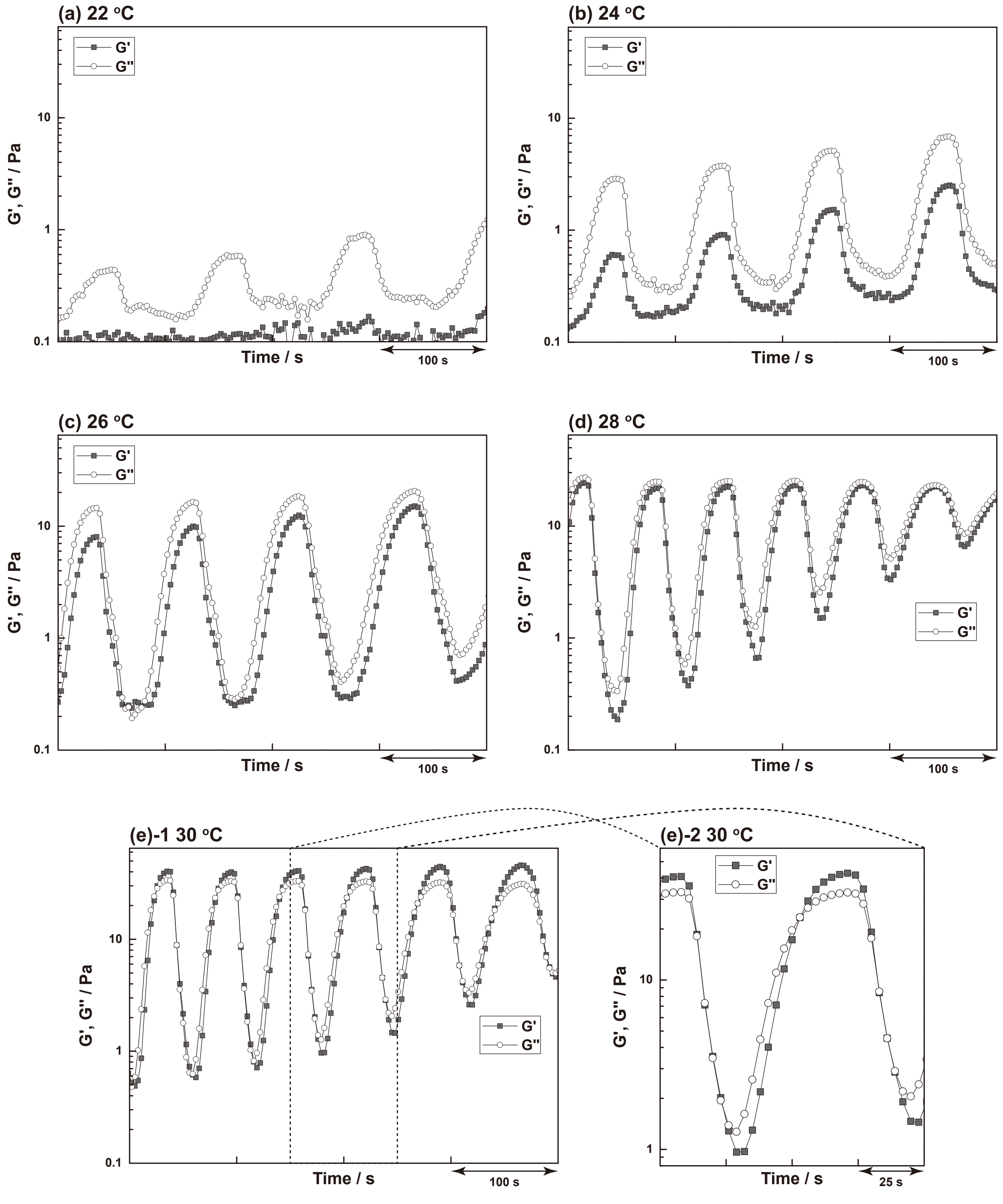

Supplementary Figure 14 Oscillating profiles of *G*' and *G*" for the ABC triblock copolymer solutions (5.0 wt%). Temperatures of each measurements were set at **(a)** 22 °C, **(b)** 24 °C, **(c)** 26 °C, **(d)** 28 °C, and **(e)** 30 °C. The BZ substrates in the feed for the measurements were composed as follows: [HNO_3] = 0.81 M, [$NaBrO_3$] = 0.1 M, [MA] = 0.04 M, γ = 2.0 %, f = 1.0 Hz.

Supplementary Table 1　Characterization results for the ABC triblock copolymer synthesized in this study.

Polymer Formula [1), 2)]	M_n [2)] / kDa	PDI [3)]
$P(NI_{0.85}$-*r*-$BA_{0.15})_{107}$-*b*-$PDMAAm_{414}$-*b*-$P(NI_{0.96}$-$NA_{0.01}$-$RuNA_{0.03})_{180}$	12-41-21	1.35

[1)] Abbreviations of the monomers are as follows; NI: *N*-Isopropylacrylamide (NIPAAm), BA: Butyl acrylate, DM: *N*, *N*-Dimethylacrylamide, NA: *N*-3-(Aminopropyl)methacrylamide (NAPMAm), RuNA: $Ru(bpy)_3$ moiety attached to the amino group of NAPMAm via an amide bond. Numbers following the abbreviations of monomer indicate the number of repeating units. [2)] Calculated from ^{1}H-NMR. [3)] Calculated from GPC.

Supplementary Table 2　The BZ substrates in feed for the measurements of Supplementary Fig. 12.

**Figure**	[Polymer] / wt%	[HNO_3] / M	[$NaBrO_3$] / M	[MA] / M
**(a)**	0.1	0.60	0.1	0.04
**(b)**	0.1	0.81	0.1	0.04
**(c)**	5.0	0.70	0.1	0.04
**(d)**	5.0	0.90	0.1	0.04

Supplementary Table 3　Periods of each process in the course of BZ reaction of the 0.1 wt% polymer solution of Supplementary Fig. 12.

[HNO_3] / M	Process A / s	Process B / s	Process C / s	Total Period / s
0.60	17	17	32	66
0.81	5	19	29	53

Supplementary Table 4　Periods of each process in the course of BZ reaction of the 5.0 wt% polymer solution of Supplementary Fig. 12.

[HNO_3] / M	Process A / s	Process B / s	Process C / s	Total Period / s
0.70	9	44	32	85
0.90	3	44	43	90
